# Characterising the activity, lifestyle behaviours and health outcomes of UK university students: an observational cohort study with a focus on gender and ethnicity

**DOI:** 10.1186/s12889-024-20911-0

**Published:** 2024-12-18

**Authors:** Matthew J. Savage, Eleanor L. Procter, Daniele Magistro, Philip J. Hennis, James Donaldson, Anika Leslie-Walker, Bethany A. Jones, Ruth M. James

**Affiliations:** 1 SHAPE Research Group, School of Science and Technology, Nottingham Trent University, Nottingham, UK; 2 Department of Psychology, Nottingham Trent University, Nottingham, UK; 3 Diabetes Research Centre, University of Leicester, Leicester, UK; 4 Department of Cardiovascular Sciences, University of Leicester, Leicester, UK

**Keywords:** Student, Lifestyle, Behaviour, Health, Gender diversity, Ethnicity

## Abstract

**Background:**

Health-related outcomes and behaviours in university students are known to be poor relative to the general population. The substantial contextual shifts related to the COVID-19 pandemic, combined with increased numbers of students from minoritised ethnicity backgrounds and presenting as trans and gender diverse (TGD), means that up-to-date information is unavailable. The primary aim of this study was therefore to characterise the current movement, dietary and lifestyle behaviours, mental health, and Body Mass Index (BMI) of UK university students and assess differences between genders and ethnic groups.

**Methods:**

An online, self-report survey was administered across three years (2021–2023). Three independent cohorts of university students’ (*n* = 6,327) completed the survey on four key topic areas. One-way ANOVAs were used to assess differences between genders (men, women, TGD), and independent samples t-tests were used to assess differences between ethnic groups (White, Minoritised Ethnicity).

**Results:**

30% of students were not meeting physical activity guidelines, 54% were sedentary for ≥ 6 h·d^− 1^, 83% had poor diet quality, 51% were in high or increased risk groups for alcohol consumption, 18% experienced terrible or poor sleep quality, and 32% were overweight or obese. Gender differences were present for all variables other than walking physical activity (WPA) (*P* < 0.05), with men having better mental health and engaging in healthier movement and sleeping behaviours, whereas women had more healthful dietary and drinking behaviours, and TGD students had poorer outcomes compared to cis-gender students in most domains. Differences between White and minoritised ethnicity students were present for all variables other than sedentary behaviour, diet quality, WPA and BMI (*P* < 0.05); students of minoritised ethnicity engaged in better movement, drinking and sleep behaviours in addition to having more positive mental health than White students.

**Conclusion:**

The findings of the current study provide an update on the landscape of UK university students’ health and health-related behaviours. Overall, health-related outcomes and behaviours are poor in this population and these data suggest that gender and ethnicity play a role in determining students’ health and health-related behaviours. Therefore, these factors should be considered when developing strategies to promote healthy living in the context of higher education.

## Introduction

In 2020, 2.8 million students were enrolled on higher education courses in the UK [1]. University students now represent a substantial proportion of young people within the UK, with 50% of school leavers continuing to higher education [2, 3]. Worryingly, the transition from secondary to higher education has previously been shown to negatively influence outcomes of health and related behaviours [4]. Indeed, high numbers of university students were consuming a problematic diet [5], undertaking sub-optimal amounts of physical activity [5], engaging in high levels of sedentary behaviour (SB) [6], and partaking in binge drinking [7]. Additionally, 52% of first year university students in England experience substantial weight gain (> 0.5 kg) [8] and living on-campus leads to greater increases in body mass compared to living off-campus (1.65 kg vs. 0.13 kg) [9]. Furthermore, 1 in 5 university students have a current mental health diagnosis and 30% of UK university students suffer from clinically low mental wellbeing (MWB) [5, 10]. Taken together, these data indicate that the health and health behaviours of UK university students are sub-optimal, and this could negatively impact future physical and mental health of a large proportion of the population [11, 12].

Importantly, the COVID-19 pandemic has exacerbated this issue further. Studies demonstrate that physical activity decreased, and sedentary behaviour (SB) increased, during different stages of the pandemic [13, 14]. Additionally, student’s sleeping patterns and eating behaviours were negatively impacted during lockdown periods [15, 16], and students experienced significant weight gain and impaired mental health [13, 14, 17]. Furthermore, since the removal of restrictions, some evidence suggests that health-related behaviours remain impaired compared to pre-lockdown levels in children and adults [18], and levels of anxiety remain higher than pre-pandemic periods in university students [19]. This is particularly concerning given that early adulthood is a critical time for establishing health-related habits and behaviours that are sustained throughout the lifespan, and that negative health behaviours adopted when young can accelerate the occurrence of morbidity in later life [11]. As such, if the adverse trend in students’ health behaviours continues, it could exacerbate the strain on already overstretched public health systems throughout the UK [20]. The issue of poor student health is therefore more pertinent than ever.

To begin addressing this problem, universities require up-to-date information surrounding the current health status of the general student population. Within this, recent literature (spanning 2014–2023) from across the globe has identified considerable differences between genders and ethnic groups in relation to outcomes of students’ movement and nutrition behaviours, psychological markers, and anthropometric outcomes [21–31]. For instance, students who are men have been shown to engage in more healthful movement behaviours than women, whereas women observed better dietary habits [30]. Additionally, students of minoritised ethnicity are more likely to have poorer mental health outcomes compared to their White counterparts [31]. It is important to note that the proportion of people presenting as trans and gender diverse (TGD) in the UK general population has increased dramatically in recent years [32] and similar increases have been observed in students [1]. Furthermore, the number of students from ethnically diverse and international backgrounds are at an all-time high [1], greatly accelerating the diversification of the student demographic. As such, currently, relatively little is known about health outcomes and associated behaviours in these populations. It is therefore vital that up-to-date data are available to identify current gender and ethnic disparities in relation to student health, in order to aid in the development of effective strategies to improve outcomes of health and related behaviours in students.

The primary aim of this study was to characterise the current movement, dietary and lifestyle behaviours, mental health and Body Mass Index (BMI) of UK university students. The secondary aim was to assess differences between genders and ethnic groups.

## Methods

### Participants and setting

**Fig. 1:**
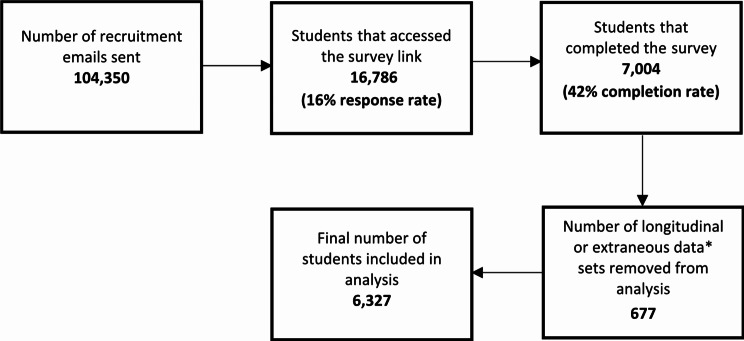
Recruitment data for participation in the survey (2021–2023). Examples of extraneous data include data that was removed due to entering an incorrect identification code, and data that was outside of the limits considered to be possible within the survey scale (i.e., PA values ≥ 16 h in accordance with the IPAQ scoring guidelines) [40]

Students from a single, large, ‘post-92’ university in the East Midlands of the UK were recruited via email to complete a self-report online survey during the first term of one of three consecutive academic years (2021-22; 2022-23; or 2023-24) when all government-imposed COVID-19 restrictions had been lifted and teaching modalities had stabilised. A total of 6,327 students comprise the data set analysed in this study. The specific details of the recruitment process are shown in Fig. 1. The population within this study had a high representation of minoritised ethnicity (33.6% vs. 23.6%) and women (66.2% vs. 57.0%) relative to UK student population norms [33, 34]. Prior to completing the survey, participants provided informed consent. All data were pseudo-anonymised and remained confidential throughout. The study was conducted in accordance with STROBE guidelines [35] and ethical approval was granted by the School of Science and Technology Non-invasive Ethics Committee of Nottingham Trent University (application ID: 19/20–76).

### Survey design

The survey contained socio-demographic questions (8 items; Table 1) and a health history question (Do you suffer from any diagnosed long-term health condition(s)?; 1 item). The survey also contained two validated scales, Cohen’s Perceived Stress Scale (PSS) [36] and the Short Warwick-Edinburgh Mental Wellbeing Scale (S-WEMWBS). The PSS uses a 5-point Likert scale (0=‘Never’ to 4=‘Very often’) with possible total scores ranging from 0 to 40, where higher scores are indicative of greater levels of PS. The S-WEMWBS uses a similar 5-point Likert scale (1= ‘None of the time’ to 5=‘All of the time’) with possible total scores ranging from 7 to 35, where higher scores indicate better mental wellbeing. Both scales have been previously validated in UK students (Cronbach’s alpha = 0.89 & Composite reliability (ρc) = 0.88 respectively) [37, 38]. The survey also included the United States Alcohol Use Disorders Identification Test – Consumption (USAUDIT-C), a 3 item scale to identify risky drinking behaviour where each item is scored on a 6-point Likert scale (0=‘Never’ or ‘1 drink’ to 5=‘Daily’ or ‘10 or more drinks’) and scores were calculated from the sum of each item. Scores range from 0 to 18 whereby a score of ≥ 7 is a positive risk indicator in women and a score of ≥ 8 is a positive risk indicator in men, however there are no guidelines on scores indicating positive risk for TGD individuals and as such these students were not included in categorical analysis for this variable. The USAUDIT-C has previously been validated in university students [39]. The International Physical Activity Questionnaire – Short Form was also included in the survey which enables the calculation of moderate (MPA), vigorous (VPA), and walking (WPA) intensity physical activity as well as time spent sitting on weekdays during the previous seven days (IPAQ -SF) [40]. Responses were scored in alignment with the IPAQ protocol (www.ipaq.ki.se) to identify the amount MVPA undertaken per week, and this has previously been validated in university students [41]. A single-item sleep quality scale (SQS) [42] and a short-form food frequency questionnaire (SFFQ) [43] were also included in the survey. The single-item SQS evaluates subjective feelings of night-time sleep quality during the previous seven days using a 10-point Likert scale with 0 being ‘terrible’ and 10 being ‘excellent’. The SFFFQ is a 27-item questionnaire whereby participants were asked how often they consumed each item on average during a typical week. Participants were asked to select one of eight frequency categories ranging from ‘rarely or never’ to ‘five or more times per day’ and from there a diet quality score (DQS) was calculated as per the SFFFQ protocol [43]. Although not in university students, both scales have been previously validated [42, 43].

**Table 1:** Participant information (*n* = 6,327; data presented as *n* (%) or M ± SD)

	Total	***Men***(***n*** = 1968)	***Women*** (***n*** = 4186)	***TGD*** (***n*** = 164)	***White*** (***n*** = 4146)	***Minoritised ethnicity*** (***n*** = 2125)
**Age (years)**						
18–21	4043 (63.9)	1229 (62.4)	2677 (64.0)	130 (79.3)	3006 (72.5)	1010 (47.5)
22–25	1204 (19.0)	378 (19.2)	799 (19.1)	26 (15.9)	667 (16.1)	522 (24.6)
26–35	743 (11.7)	262 (13.3)	473 (11.3)	7 (4.3)	292 (7.0)	439 (20.7)
35+	337 (5.3)	99 (5.0)	237 (5.7)	1 (0.6)	181 (4.4)	154 (7.2)
**Gender**						
Men	1968 (31.1)	-	-	-	1203 (29.0)	747 (35.2)
Women	4186 (66.2)	-	-	-	2797 (67.5)	1352 (63.6)
Non-binary	110 (1.7)	-	-	-	93 (2.2)	17 (0.8)
Trans women	10 (0.2)	-	-	-	9 (0.2)	1 (0.1)
Trans men	28 (0.4)	-	-	-	25 (0.6)	3 (0.1)
Other	16 (0.3)	-	-	-	13 (0.3)	2 (0.1)
Not specified	9 (0.1)	-	-	-	6 (0.1)	3 (0.1)
**Ethnicity**						
White	4146 (65.5)	1203 (61.1)	2797 (66.8)	140 (85.4)	-	-
Mixed	269 (4.3)	86 (4.4)	175 (4.2)	8 (4.9)	-	-
Asian	1128 (17.8)	399 (20.3)	717 (17.1)	10 (6.1)	-	-
Black	572 (9.0)	213 (10.8)	356 (8.5)	2 (1.2)	-	-
Other	156 (2.5)	49 (2.5)	104 (2.5)	3 (1.8)	-	-
Not specified	56 (0.9)	18 (0.9)	37 (0.9)	1 (0.6)	-	-
**Height (m)**	1.68 ± 0.11	1.75 ± 1.3	1.65 ± 0.8	1.67 ± 0.9	1.69 ± 1.1	167 ± 1.1
**Body Mass (kg)**	68.2 ± 16.2	77.5 ± 16.1	63.3 ± 13.7	66.4 ± 20.0	68.9 ± 16.3	66.9 ± 15.8
**BMI categories**						
Underweight (< 18.5)	430 (6.8)	85 (4.3)	323 (7.7)	21 (12.8)	267 (6.4)	159 (7.5)
Healthy weight (18.5–24.9)	2714 (42.9)	804 (40.9)	1863 (44.5)	44 (26.8)	1820 (43.9)	868 (40.8)
Overweight (25.0-29.9)	942 (14.9)	434 (22.1)	491 (11.7)	16 (9.8)	537 (13.0)	398 (18.7)
Obese (30-39.9)	488 (7.7)	213 (10.8)	264 (6.3)	10 (6.1)	325 (7.8)	160 (7.5)
Severely obese (≥ 40.0)	76 (1.2)	34 (1.7)	37 (0.9)	5 (3.0)	53 (1.3)	23 (1.1)
Not specified	1677 (26.5)	398 (20.2)	1208 (28.9)	68 (41.5)	1144 (27.6)	517 (24.3)
**University year**						
Year 1	1800 (28.4)	560 (28.5)	1173 (28.0)	63 (38.4)	1255 (30.3)	537 (25.3)
Year 2	1396 (22.1)	412 (20.9)	946 (22.6)	36 (22.0)	1036 (25.0)	352 (16.6)
Year 3	1305 (20.6)	398 (20.2)	872 (20.8)	34 (20.7)	1003 (24.2)	291 (13.7)
Year 4	298 (4.7)	95 (4.8)	197 (4.7)	6 (3.7)	248 (6.0)	48 (2.3)
Foundation	84 (1.3)	35 (1.8)	46 (1.1)	3 (1.8)	55 (1.3)	28 (1.3)
PG Master’s Degree (or equivalent)	1219 (19.3)	407 (20.7)	796 (19.0)	14 (8.5)	373 (9.0)	823 (38.7)
PhD	118 (1.9)	23 (1.2)	89 (2.1)	6 (3.7)	90 (2.2)	26 (1.2)
Other/Not specified	107 (1.7)	38 (1.9)	67 (1.6)	2 (1.2)	86 (2.1)	20 (0.9)
**Self-reported pre-existing mental health condition**						
None/Not specified	5426 (85.8)	1823 (92.6)	3492 (83.4)	103 (62.8)	3415 (82.4)	1958 (92.1)
Any mental health condition	901 (14.2)	145 (7.4)	694 (16.6)	61 (37.2)	731 (17.6)	167 (7.9)

Question not in the survey or not relevant for the category (-)

### Data interpretation

Descriptive data are reported in the form of mean ± one standard deviation and percentages are as a proportion of those who completed the relevant question in the questionnaire. Self-reported BMI was calculated using the following equation: $\:\frac{body\:mass\:\left(kg\right)}{{height\:\left(m\right)}^{2}}$

Students were categorised by BMI using the following guidelines from the UK [44]: Underweight = < 18.5 kg/m^2^; Healthy weight = 18.5–24.9 kg/m^2^; Overweight = 25.0–29.9 kg/m^2^; Obese = 30.0–39.9 kg/m^2^; Severely obese = ≥ 40.0.

To characterise the current movement, diet and lifestyle behaviours, mental health, and BMI of university students’, data collected from each year were pooled to remove the influence of time and create a single cross-sectional data set. For the purposes of these analyses, gender was clustered into three categories: men, women, and TGD (those who experience incongruence between their sex assigned at birth and gender identity) [45]. Additionally, ethnicity was clustered into two categories: White and minoritised ethnicity (nuanced categories for TGD and minoritised ethnicity are provided in Table 1). Where participants’ did not specify a gender or ethnicity, they were included in total population data but were not included in analysis between gender or ethnic groups.

A one-way ANOVA was conducted to assess differences between gender categories. This test was used despite variables violating the assumption of normal distribution as no non-parametric alternative is currently widely accepted when the sample size is substantially larger than 30 [46, 47], and one-way ANOVA’s are robust to violations of normality [48]. Variance between groups was assessed using Levene’s test and variables were considered homogenous if the value was not significant (*P* > 0.05). Mauchly’s test of sphericity was implemented, and sphericity was assumed if the test was not significant (*P* > 0.05). Where violated, the Greenhouse-Geisser correction was applied. Additionally, to assess for effect size, partial eta squared (*η*_*p*_^*2*^) was employed to calculate the magnitude of differences between genders with the parameters set as follows: (0.02–0.12 = small effect; 0.13–0.25 = medium effect; >0.26 = large effect) [49]. Post-hoc tests were conducted using the Bonferroni correction to quantify whether differences between genders were significant (*P* < 0.05). Differences between ethnic groups were assessed using an independent samples t-test. To assess effect sizes, Cohen’s d (*d*) was used with the following classifications: trivial effect (< 0.2) small effect (≥ 0.2), medium effect (≥ 0.5), and large effect (≥ 0.8) [50]. Significance was set at *P* < 0.05 and all data were analysed using IBM SPSS Statistics (SPSS V. 28.0; Chicago, IL).

## Results

The socio-demographic characteristics of the 6,327 participants who completed the survey are described in Table 1. A flow chart outlining stages of participant recruitment for the current study is displayed in Fig. 1.

### Movement behaviours

Mean MVPA in 3,240 students, and sedentary time in 2,749 students are presented in Table 2. The prevalence of students not meeting physical activity guidelines (≥ 150 min/week of MVPA) and spending ≥ 6 h per day engaging in SB are displayed in Table 3.

**Table 2:** Pooled cross-sectional data for all variables (*n* = 6,327)

	Mean ± SD
**Movement behaviours**	
MVPA (mins/week) (*n* = 3,240)	386 ± 392
SB (mins/week) (*n* = 2,749)	1898 ± 1365
MPA (mins/week) (*n* = 2,089)	256 ± 296
VPA (mins/week) (*n* = 2,710)	285 ± 261
WPA (mins/week) (*n* = 3,710)	459 ± 415
**Diet and lifestyle**	
DQS (*n* = 6,327)	9.8 ± 1.8
USAUDIT-C (*n* = 5,469)	6.8 ± 3.4
SQS (*n* = 6,326)	5.8 ± 2.3
**Mental health**	
S-WEMWBS (*n* = 6,327)	20.9 ± 3.9
PSS (*n* = 6,314)	20.5 ± 6.7
**BMI (kg/m**^**2**^) (***n*** **= 4**, **650)**	24.0 ± 5.4

* Variance in response rate reported for movement and drinking behaviours are due to IPAQ and USAUDIT-C data processing guidance respectively, and for BMI it is where participants reported not knowing their height and/or weight

**Table 3:** Prevalence of risky health behaviours, poor mental health, and overweight and obesity (*n* = 6,327; data presented as % (n))

	Overall prevalence	Prevalence by gender			Prevalence by ethnicity	
		Men	Women	TGD	White	Minoritised ethnicity
**Moderate to vigorous physical activity (MVPA)**						
Not meeting guidelines (< 150 min per week)	30.0 (969)	21.1 (264)	35.6 (674)	44.9 (31)	30.3 (691)	29.3 (270)
**Sedentary behaviour (SB)**						
≥ 6 h per day engaging in sedentary behaviour	53.8 (1479)	50.7 (546)	54.9 (882)	79.0 (49)	56.3 (1042)	48.3 (424)
**Diet quality (DQS)**						
Unhealthy diet (score < 12)	82.9 (5248)	83.6 (1645)	82.3 (3447)	89.6 (147)	82.4 (3415)	84.0 (1785)
**Alcohol drinking behaviour (USAUDIT-C)**						
High risk (score of ≥ 7 for women and ≥ 8 for men)	50.5 (2761)	48.7 (814)	53.3 (1947)	-	59.1 (2317)	28.9 (434)
**Sleep quality (SQS)**						
Terrible, poor, or fair sleep (score of 0–6)	56.1 (3550)	50.6 (995)	57.9 (2422)	77.8 (7)	57.5 (2386)	53.0 (1126)
**Mental wellbeing (MWB)**						
Low mental wellbeing (score of 7.0-19.5)	39.8 (2517)	29.4 (584)	43.5 (1819)	65.2 (107)	42.0 (1742)	35.5 (2125)
**Perceived stress (PSS)**						
High perceived stress (score of 27–40)	18.3 (1157)	10.0 (197)	21.3 (891)	41.1 (67)	20.7 (858)	13.6 (288)
**Prevalence of overweight & obesity**						
BMI ≥ 25.0	32.4 (1506)	43.4 (681)	26.6 (792)	32.3 (31)	30.5 (915)	6.1 (581)

No categorical stipulation for TGD students (-)

**Fig. 2:**
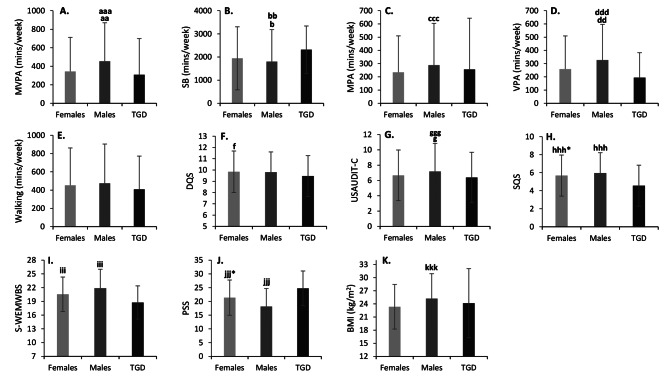
Displays the differences between genders for the reported variables ^aaa^ indicates *P* < 0.001 compared to women. ^aa^ indicates *P* < 0.01 compared to TGD students. ^bb^ indicates *P* < 0.01 compared to TGD students. ^b^ indicates *P* < 0.05 compared to women. ^ccc^ indicates *P* < 0.001 compared to women. ^ddd^ indicates *P* < 0.001 compared to women. ^dd^ indicates *P* < 0.01 compared to TGD students. ^f^ indicates *P* < 0.05 compared to TGD students. ^ggg^ indicates *P* < 0.001 compared to women. ^g^ indicates *P* < 0.05 compared to TGD students. ^hhh^ indicates *P* < 0.001 compared to women & TGD students. ^hhh*^ indicates *P* < 0.001 compared to TGD students. ^iii^ indicates *P* < 0.001 compared to women & TGD students. ^jjj^ indicates *P* < 0.001 compared to women & TGD students. ^jjj*^ indicates *P* < 0.001 compared to TGD students. ^kkk^ indicates *P* < 0.001 compared to women

A main effect of gender was observed for both MVPA (*P* < 0.001) and SB (*P* = 0.002) with small and trivial effect sizes (*η*_*p*_^*2*^ = 0.02 & *η*_*p*_^*2*^ = 0.01 respectively). On average, men participated in substantially greater amounts of MVPA (454 ± 415 min/week) compared to women (344 ± 369 min/week; *P* < 0.001) and TGD students (309 ± 390 min/week; *P* < 0.01; Fig. 2A), but there was no difference between women and TGD students (*P* = 1.00; Fig. 2A). Men engaged in less SB (1804 ± 1361 min/week) than women (1944 ± 1381 min/weekday; *P =* 0.03), and TGD students (2318 ± 1022 min/week; *P* = 0.01), but again there was no difference between women and TGD students (*P* = 0.10; Fig. 2B).

**Fig. 3:**
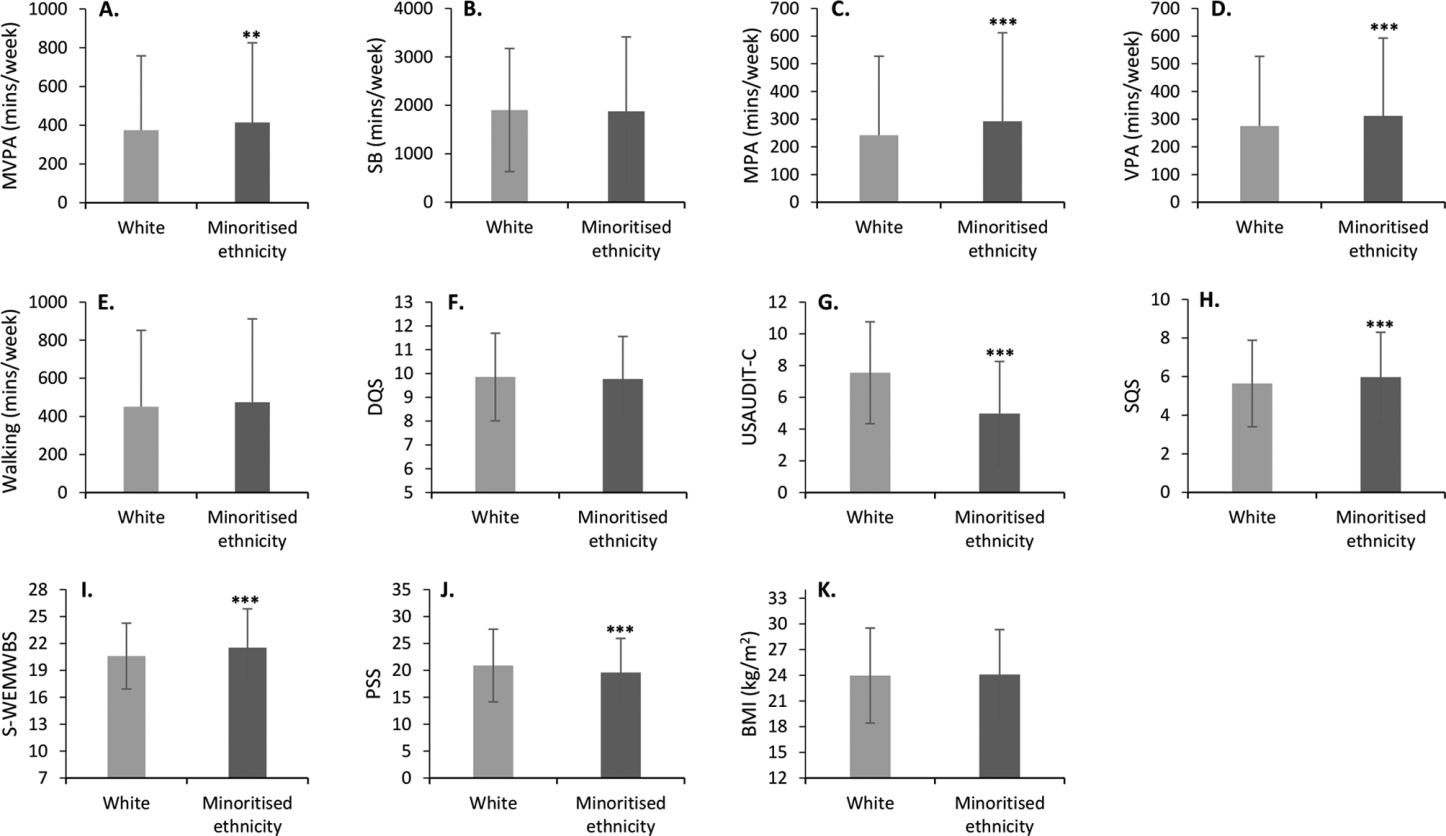
Displays the differences between ethnic groups for the reported variables. *** indicates *P* < 0.001; ** indicates *P* < 0.01; * indicates *P* < 0.05 compared to White students

When separated by ethnicity, students of a minoritised ethnicity participated in greater amounts of MVPA (423 ± 422 min/week) compared to White students (374 ± 375 min/week; *t*=-3.27; *P* = 0.002) with a trivial effect size (*d*=-0.1; Fig. 3A). However, no differences in sedentary behaviour were observed between students of a minoritised ethnicity (1874 ± 1537 min/week) and White students (1902 ± 1270 min/week; *t* = 0.50; *P* = 0.64; Fig. 3B).

Descriptive data surrounding different intensity domains of PA (MPA, VPA & WPA) are provided in Table 2. A main effect of gender was observed for MPA (*P* < 0.001) and VPA (*P* < 0.001) with trivial and small effect sizes (*η*_*p*_^*2*^ = 0.01 & *η*_*p*_^*2*^ = 0.02 respectively), but no effect of gender was found for WPA (*P* = 0.17; Fig. 2E). Men engaged in greater amounts of MPA (288 ± 316 min/week) than women (234 ± 275 min/week; *P* < 0.001), but not TGD students (256 ± 387 min/week; *P* = 1.00), and no differences existed between women and TGD students (*P* = 1.00; Fig. 2C). Men also engaged in greater amounts of VPA (327 ± 270 min/week) than both women (258 ± 251 min/week; *P* < 0.001) and TGD students (194 ± 189 min/week; *P* < 0.01), but again no differences existed between women and TGD students (*P* = 0.32; Fig. 2D).

When separated by ethnicity, students of a minoritised ethnicity engaged in greater amounts of MPA (292 ± 320 min/week) and VPA (312 ± 281 min/week) compared to White students (242 ± 285 min/week, *t*=-3.37, *P* < 0.001 & 275 ± 252 min/week, *t*=-3.15, *P* < 0.001 respectively; Fig. 3C and D) with small and trivial effect sizes (*d*=-0.2, *d*=-0.1). However, there was no difference in WPA between White students (450 ± 402 min/week) and students of a minoritised ethnicity (474 ± 438 min/week, *t* = 0.47, *P* = 0.64; Fig. 3E).

### Diet and lifestyle

The mean DQS in 6,327 students, USAUDIT-C score in 5,469, and SQS in 6,326 students are presented in Table 2. The prevalence of students with poor diet quality (score < 12), hazardous drinking behaviour (score of ≥ 7 for women and ≥ 8 for men), and terrible, poor, or fair sleep quality (score of 0–6) are displayed in Table 3.

A main effect of gender was observed for DQS (*P* = 0.03), USAUDIT-C *P* < 0.001), and SQS (*P* < 0.001) with trivial effect sizes (*η*_*p*_^*2*^ = 0.001, *η*_*p*_^*2*^ = 0.01 & *η*_*p*_^*2*^ = 0.01 respectively). Mean DQS was not different between men (9.8 ± 1.8) and women (9.9 ± 1.8; *P* = 1.00) or men and TGD students (9.5 ± 1.8; *P* = 0.06; Fig. 2F). However, women had a greater DQS than TGD students (*P* = 0.03) On average, men had a higher USAUDIT-C score (7.2 ± 3.7) compared to women (6.7 ± 3.3;*P* < 0.001) and TGD students (6.4 ± 3.3; *P* = 0.03), but no differences existed between women and TGD students (*P* = 1.00; Fig. 2G). Furthermore, men had a higher SQS (6.0 ± 2.2) than women (5.7 ± 2.3; *P* < 0.001), and both men and women had a higher SQS than TGD students (4.6 ± 2.3; *P* < 0.001; Fig. 2H).

When separated by ethnicity, no differences were observed in DQS between students of a minoritised ethnicity (9.8 ± 1.8) and White students (9.9 ± 1.8; *t* = 1.73; *P* = 0.08; Fig. 3F). Additionally, White students had a greater USAUDIT-C score (7.6 ± 3.2) compared to students of a minoritised ethnicity (5.0 ± 3.3; *t* = 25.98; *P* < 0.001) with a large effect size (*d* = 0.8; Fig. 3G). Finally, SQS was higher in students of a minoritised ethnicity (6.0 ± 2.3) compared to White students (5.6 ± 2.2; *t*=-5.40; *P* < 0.001) with a trivial effect size (*d*=-0.1; Fig. 3H).

### Mental health

The mean S-WEMWBS score in 6,327 students, and mean PSS score in 6,314 students is presented in Table 2. The prevalence of students with low MWB (score ≤ 19.5) and high PSS (score of 27–40) is displayed in Table 3.

A main effect of gender was observed for both MWB (*P* < 0.001) and PSS (*P* < 0.001) with small effect sizes (*η*_*p*_^*2*^ = 0.03 & *η*_*p*_^*2*^ = 0.06 respectively). On average, men (21.9 ± 4.1) had a higher S-WEMWBS score compared to women (20.6 ± 3.8; *P* < 0.001) and both men and women had higher S-WEMWBS scores compared to TGD participants (18.8 ± 3.6; *P* < 0.001; Fig. 2I). Men had a lower PSS score (18.2 ± 6.5) than women (21.4 ± 6.4; *P* < 0.001) and both men and women had lower PSS scores compared to TGD students (24.8 ± 6.3; *P* < 0.001; Fig. 2J).

When separated by ethnicity, students of a minoritised ethnicity had a higher S-WEMWBS score (21.5 ± 4.3) compared to White students (20.6 ± 3.7; *t*=-8.54; *P* < 0.001) with a small effect size (*d* = 0.2; Fig. 3I). Additionally, students of a minoritised ethnicity had a lower PSS score (19.6 ± 0.7) compared to White students (20.9 ± 6.7; *t* = 7.43; *P* < 0.001) with a small effect size (*d* = 0.2; Fig. 3J).

### BMI

The mean BMI of 4,650 students is presented in Table 2. The prevalence of students classified as having overweight/obesity (BMI ≥ 24.9 kg/m^2^) is displayed in Table 3.

A main effect of gender was observed for BMI (*P* < 0.001) with a small effect size (*η*_*p*_^*2*^ = 0.03). Men had a higher BMI (25.2 ± 5.7 kg/m^2^) than women (23.3 ± 5.1 kg/m^2^; *P* < 0.001) but there were no differences between men and women, and TGD students (24.2 ± 7.9 kg/m^2^; *P* > 0.05; Fig. 2K).

No differences were observed between White students (24.0 ± 5.6 kg/m^2^) and those of a minoritised ethnicity (24.1 ± 5.2 kg/m^2^; *t*=-0.69; *P* = 0.49; Fig. 3K).

## Discussion

The findings of the current study indicate that large proportions of UK university students engage in sub-optimal health-related behaviours which could increase the risk of poor health outcomes in the future. Additionally, the results demonstrate that both gender and ethnicity substantially influence health and health-related behaviours in students.

### Movement behaviours

Overall, mean MVPA in university students was substantially greater than the government guidance of 150 min of moderate to vigorous intensity exercise per week [51]. However, we show that 30.0% of students were not meeting this target. While it is initially encouraging that this is substantially lower than the data from previous literature which showed that 60% of UK students were insufficiently active [5], 30.0% is still higher than age-matched people within the UK general population in 2018 (23%) [52]. The apparent reduction in the proportion of students not sufficiently active (60–30%) appears at first sight to be promising. However, changes to UK guidance in 2019 [53] may be, in part, responsible for this. Furthermore, the data reported by [5] were based on a substantially smaller cohort and were gathered using a questionnaire validated in elderly people, not students. It is of course possible that the reduction in those not meeting guidelines also stems from an increased health awareness following the COVID-19 pandemic [54].

Additionally, the current study demonstrates that mean SB was 6.3 h per day, which is higher than the daily threshold of 6 h SB, beyond which there are suggested to be negative long term health outcomes [55]. Indeed, 53.8% of students were engaging in sedentary activities for > 6 h per day, which is greater than the 49.1% proposed in a previous global review of student’s sedentary behaviour in 2020 [56]. Taken together, these findings are concerning given the detrimental effects insufficient PA and excessive SB are known to have on health [57, 58].

This study adds further evidence to literature demonstrating that men undertake more PA and less SB than women [5, 30, 58]. However, less is known about the TGD student population, and the current study indicates that these students engage in lower levels of MVPA than men but comparable levels to women. Additionally, when split by intensity, TGD students only engage in less VPA than men but undertake comparable levels to their cisgender peers in all other intensity domains. Furthermore, our data indicate that TGD students engage in substantially greater periods of SB than their peers who were men, but not women. Explanations for the differences shown between TGD and cisgender students movement behaviours are beyond the scope of this study but, may be due to reduced social support and increased negative physical self-perceptions in the gender-diverse population that discourage this population from engaging in vigorous physical activity [25].

The current study indicates that students of a minoritised ethnicity engage in greater amounts of PA (across all intensity domains other than walking) and similar amounts of SB compared to White students, which contrasts previous UK-based evidence from 2019 to 2021 [59, 60]. Previously, differences were purported to be due to socioeconomic background, whereby minoritised ethnic groups may not be able to access fitness facilities due to increased financial burden [61, 62]. However, institutional barriers such as access to preferred activities and awareness of opportunities have also been cited as barriers to PA in minority ethnic women [63]. The findings of the current study may therefore reflect the high accessibility to facilities and equipment at the host institution, increasing the opportunity for students from minoritised ethnicity backgrounds to engage in PA. Whilst our findings may reflect positive change, the diversification of students undertaking tertiary education continues to accelerate, and the wider consensus remains that minoritised ethnicity students engage in poorer movement behaviours [59, 60].

### Diet and alcohol

The present study revealed 82.9% of students had an ‘unhealthy diet’ consistent with data from UK’s aged-matched population in 2018 [64]. However, 50.5% of students in the current study demonstrated hazardous drinking behaviour which is considerably larger than the 28% proposed in the general UK population in 2021 [65]. This is consistent with previous literature from 2010 indicating that 56% of UK students binge drink at least once a week [7], and 2011 showing that 52% are classified as hazardous or harmful drinkers [66], suggesting little change over the past decade. Previously, it has been suggested that poor dietary behaviours in students develop due to intrapersonal and institutional barriers such as poor cooking ability and knowledge [67], financial constraints [68], limited food availability on campus and social pressure [69]. Additionally, elevated alcohol consumption is often viewed as an integral part of the university experience [70] and students may be socially excluded if they abstain from alcohol [71]. However, poor dietary and drinking behaviours can have negative implications for future health with increased risk of developing NCDs, weight gain, alcohol dependence, and premature morbidity [72–74]. As such, universities should aim to implement previously successful interventions, or explore developing novel initiatives centred around altering environmental factors (e.g., point of purchase promotions) whilst considering intrapersonal influences (e.g., nutritional knowledge) as a means of improving nutrition behaviours in university students [75–77].

Although previous literature from Spain in 2012 has suggested that women tend to have a higher diet quality than men [78], the current study showed no difference in DQS between men and women, but women had a higher DQS than TGD students. In line with previous literature [79], men consumed more alcohol than women and TGD students, possibly due to men’ greater engagement with promotions encouraging alcohol use in nightlife settings [80] and more widely accepted social norms [81]. Those from minoritised ethnic backgrounds consumed substantially less alcohol than their White counterparts, again in line with previous literature, and likely due to differences in religious, cultural and societal influences [66, 79]. This study found no difference in DQS between White students and students of a minoritised ethnicity, in line with literature indicating that ethnicity was not associated with differences in dietary pattern behaviour in UK university students [67]. Nonetheless, previous studies have suggested that students from minoritised ethnicity backgrounds have poorer nutritional knowledge [82] and are more likely to make food choices based on cost, inconvenience, and taste rather than poor nutrient quality [83]. As such, higher education institutions should continue to develop healthy eating initiatives that aim to improve nutritional knowledge and provide cost effective, healthful food options for university students.

### Sleep quality

The current study identified that student sleep quality was similar to that of age-matched individuals in the UK [84] and aligns with previous literature indicating that over a third of students in the UK sleep less than the recommended 7 h per night due to academic timetabling and exam scheduling [85].

Gender and ethnicity may also play a role in determining sleep quality [86–91]. Indeed, the current study follows previous trends by identifying that men had the highest sleep quality, followed by women [86], while TGD students experienced the poorest sleep quality in line with previous findings [89]. Whilst the reasons for this are outside the scope of the current study, it could be related to gender differences surrounding perceived stress [92], anxiety [85] and other mental health symptoms that can influence sleep [89, 93]. Furthermore, White students experienced a lower SQS than their ethnically minoritised peers, providing contrasting evidence to previous literature [90, 91]. Whilst the reasons underpinning the above findings require further investigation, it is well-known that sufficient sleep is vital for both physical and mental health due to its key role in brain, cardiovascular and immune system function [85]. It is therefore in the interest of higher education institutions to explore methods of optimising sleep in students, taking into consideration gender and ethnicity.

### Mental health

Data from the current study indicate that on average, university students have poorer mental wellbeing and perceive themselves to be more stressed than age-matched non-students [94, 95]. This data add to a plethora of existing literature demonstrating the adverse effects of university life on student mental health in the UK [96, 97]. Given these findings, it is concerning that poor mental health in students’ is seemingly reflective of ‘normal’ student life, particularly given the well-established relationships between mental health, physical health, behaviours, and academic outcomes [98–103].

The current study also provides further evidence that gender and ethnicity may play a role in determining the mental health status of students. Specifically, men had better mental wellbeing and lower levels of perceived stress compared to women, who in turn had better MWB and lower levels of PS compared to TGD students’ findings that are supported by previous literature [24, 104]. This pattern of findings have been replicated in the wider general population [105, 106] and so it is possible that these data are reflective of current societal trends (such as using rumination as a coping style, having problematic relationships with parents and peers, increased discrimination, stigma & isolation) that suggests women [107, 108] and TGD students [104] are at greater risk of developing poorer outcomes of mental health.

Additionally, ethnically minoritised students had better MWB and lower PS compared to White students. These findings are in stark contrast to those observed within majority of previous literature demonstrating that ethnically minoritised students experience inequality in relation to accessing mental health services, which ultimately leads to poorer mental health outcomes [31]. However, in recent years there is some encouraging evidence to suggest work is being conducted to attempt to reduce this gap [109, 110]. This includes introducing ‘ethnic matching’ to ensure mental health services are culturally appropriate and reflective of the diverse student population [109, 110], and the findings of the current study may therefore positively reflect efforts to reduce ethnic disparities in relation to mental health.

### BMI

The current study indicates that mean student BMI is within the healthy range and comparable to that of age-matched young people from the UK [111]. Additionally, the prevalence of overweight or obesity in students was similar to that of normative UK data (32.4%) [111]. Whilst this shows that university life is not uniquely impacting students weight management [8, 9], it is disappointing that the commonplace weight control problems in Western society persist even within the context of higher education [112].

The current study also found that men had a higher BMI on average than women, but there was no difference between women and TGD students. The higher BMI in men may be attributable to the well-established observations that men have higher levels of skeletal muscle mass across the lifespan than women [113]. This is further supported by the findings that men engage in greater levels of physical activity than women, which may incorporate greater amounts of resistance training [114]. No differences in BMI were observed between ethnic groups. Whilst this provides contrasting evidence to that of previous literature in UK young adults [115], it may be reflective of the adequate amounts of PA (≥ 150 min of MVPA) achieved in both ethnic groups in the current study. Additionally, similar levels of SB were observed between ethnic groups which may also play a role in explaining the lack of difference in BMI.

### Strengths and limitations

The current study utilised self-reported questionnaires which may lead to inaccuracies, most notably PA levels being overestimated [116]. However, using validated survey questions minimised the potential to collect inaccurate data. Although there were no COVID-related, government-imposed restrictions at any point during data collection for this study (October 2021 – October 2023), it should be noted that the prevalence of the virus circulating in society varied during this time, with greater prevalence earlier in the study before diminishing over time. However, the methods and modality of teaching at the institute remained stable throughout the study period. Additionally, the gender and ethnic categories used in this study may mask any distinct differences between more nuanced groups. Nonetheless, the relatively low sample size within these groups would make drawing clear conclusions difficult. Furthermore, the inclusion of the IPAQ-SF allowed for quantification of walking, moderate, and vigorous PA which enabled analysis of PA in different intensity domains. Whilst it is suggested that these data are presented as METs/week, UK government guidelines surrounding PA are defined in mins/week and as such, the current study also utilises mins/week to compare against normative data and provide a more ecologically valid assessment of PA in students. These data also provide an extensive, up-to-date baseline for future studies following the cessation of COVID-related restrictions. This will ultimately aid key stakeholders in decision making when distributing resources to develop and implement interventions that aim to improve aspects of students’ health and health related behaviours. It should be noted that self-report studies of this nature are all at risk of self-selection bias based on gender and engagement with personal health [117]. However, the current study’s stratification of gender, including TGD students, is a unique and progressive approach. Furthermore, the large sample size and relatively high number of students of minoritized ethnicity also means that the results of the current study can be applied across the UK student population. Nevertheless, future studies should continue to recruit large numbers of students and aim undertake longitudinal data collection, to gain a greater understanding of trends in students health and related behaviours.

## Conclusion

Findings from the current study provide further evidence that university students have sub-optimal outcomes related to aspects of health and health-related behaviours, and that gender and ethnic differences exist within this context.

Given that universities are uniquely positioned to provide or influence their student’s movement, diet, social and educational behaviours, stakeholders should utilise these data to aid in the development of health-based initiatives targeted at specific sub-populations, in order to promote physical and mental wellbeing in an inclusive, diverse academic environment.

## Data Availability

The datasets used and/or analysed during the current study are available from the corresponding author on reasonable request.

## Abbreviations

MWB: Mental wellbeing
PS: Perceived stress
MVPA: Moderate to vigorous physical activity
MPA: Moderate physical activity
VPA: Vigorous physical activity
WPA: Walking physical activity
SB: Sedentary behaviour
DQS: Diet quality score
SQS: Sleep quality score
USAUDIT-C: United States Alcohol Use Disorders Identification Test – Consumption
BMI: Body mass index
